# Systematic analysis of different degrees of haemolysis on miRNA levels in serum and serum-derived extracellular vesicles from dogs

**DOI:** 10.1186/s12917-022-03445-8

**Published:** 2022-09-22

**Authors:** Matias Aguilera-Rojas, Soroush Sharbati, Torsten Stein, Mario Candela Andrade, Barbara Kohn, Ralf Einspanier

**Affiliations:** 1 Institute of Veterinary Biochemistry, Department of Veterinary Medicine, Freie Universität Berlin, 14163 Berlin, Germany; 2 Department of Human Anatomy, Health and Medical University Potsdam, 14471 Potsdam, Germany; 3 Small Animal Clinic, Department of Veterinary Medicine, Freie Universität Berlin, 14163 Berlin, Germany

**Keywords:** Biomarker, Dogs, Extracellular vesicles, Haemolysis, miRNA, Serum

## Abstract

**Background:**

Circulating microRNAs (miRNAs) are described as promising non-invasive biomarkers for diagnostics and therapeutics. Human studies have shown that haemolysis occurring during blood collection or due to improper sample processing/storage significantly alters the miRNA content in plasma and serum. Nevertheless, no similar research has been performed in dogs so far. We therefore investigated the effects of different degrees of haemolysis on the levels of selected miRNAs in serum and serum-derived extracellular vesicles (EVs) from dogs, by inducing a controlled in vitro haemolysis experiment.

**Results:**

The abundance of miR-16, miR-92a, miR-191, miR-451 and miR-486 was significantly sensitive to haemolysis in serum and serum-derived EVs, while other selected miRNAs were not influenced by haemolysis. Furthermore, we found that the abundance of some canine miRNAs differs from data reported in the human system.

**Conclusions:**

Our results describe for the first time the impact of haemolysis on circulating miRNAs not only in whole serum, but also in serum-derived EVs from dogs. Hence, we provide novel data for further analyses in the discovery of canine circulating biomarkers. Our findings suggest that haemolysis should be carefully assessed to assure accuracy when investigating circulating miRNA in serum or plasma-based tests.

**Supplementary Information:**

The online version contains supplementary material available at 10.1186/s12917-022-03445-8.

## Background

MicroRNAs (miRNAs) are functional short noncoding RNA molecules containing about 22 nucleotides in length and are identified as one of the fundamental regulators of gene expression [1]. By means of post-transcriptional gene silencing, miRNAs are involved in most, if not all, biological processes and therefore their dysregulation has been associated with a large number of diseases [2, 3]. The first observation that miRNAs are present in biological fluids was made by Chim et al. [4] and almost simultaneously by Lawrie et al. [5], who respectively detected miRNA species in plasma and serum samples. Since the discovery of extracellular/circulating miRNAs, research in miRNAs in all types of biofluids has steadily developed, particularly in serum and plasma. Numerous studies have reported that extracellular vesicles (EVs) such as exosomes and microvesicles, high-density lipoproteins, and ribonucleoprotein complexes are responsible for carrying circulating miRNAs. These transport mechanisms keep miRNAs protected from RNase degradation, which increases their stability and circulation time [6, 7]. Moreover, circulating miRNAs were found to be stable for more than 10 days at room temperature and at least up to 10 years if stored at − 20 °C [8]. Due to the wide range of biological functions and their observed stability in biofluids, circulating miRNAs have emerged as powerful non-invasive biomarkers of disease and other clinical conditions [9].

Serum and plasma samples are easily accessible and routinely collected in the animal practice. At the same time, they represent the most promising and best studied sources of circulating miRNAs. However, pre-analytical and analytical factors may seriously affect the profile of investigated miRNAs [10]. Cellular contamination and haemolysis can affect the abundance of circulating miRNAs detected in serum or plasma and therefore induce changes in miRNA levels not related to any biological alteration [9]. Since haemolysis often occurs during blood collection and/or sample processing, several human studies using spectrophotometry [9–12] have investigated the effects of varying degrees of haemolysis on the concentration of certain miRNAs in human blood samples. Thereby, significant changes in the abundance of specific miRNAs have been detected in plasma and serum, often in the absence of colour change visible to the eye. miR-16, miR-92a, miR-451 and miR-486, known to be present in human red blood cells (RBCs), have been confirmed as haemolysis-associated miRNAs [11]. Nevertheless, many other miRNAs have also been shown not to be influenced by haemolysis, which supports their potential role as candidates for diagnostic purposes and/or as prognostic biomarkers, even from haemolysed samples.

Despite several studies having previously investigated the effect of haemolysis on circulating miRNAs in serum and plasma in humans, no comparable research has been performed in the canine system to the authors’ knowledge. Therefore, this study aimed to assess the effects of different levels of haemolysis on the abundance levels of selected miRNAs in serum samples from dogs, by generating an artificial haemolysis series experiment. In addition, we have further investigated whether haemolysis has an impact on the levels of circulating miRNAs derived from EVs isolated via precipitation methods from haemolysed serum. Evaluating the sensitivity of circulating miRNAs to haemolysis allows for the selection of haemolysis-independent, and thus more robust, candidate miRNA biomarkers in dogs.

## Results

### Assessment of haemolysis via visual inspection

**Fig. 1:**
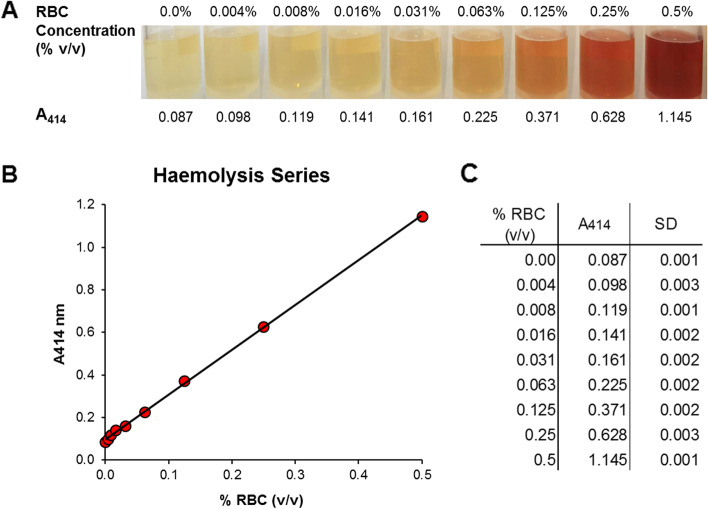
RBCs serial dilutions (1:2) in canine sera (controlled haemolysis). A Visual inspection and measurement of absorbance of haemoglobin at 414 nm (A414). B Haemolysis series showing a linear association between A414 and the increase of red/pink colouration in the serial dilutions of RBCs. C RBCs serial dilutions (% v/v) associated with their respective A414 value. Absorbance values represent mean of triplicate measurements ± SD. RBCs: red blood cells

Haemolysis was assessed via visual inspection of serial dilutions of RBCs against a white background (Fig. 1A). This allowed the detection of an increasing red/pink colouration in serum samples, starting from dilution 0.063% (v/v) RBCs. A more intense red colour was gradually observed from dilution 0.25% RBCs. Variations in colour between dilutions 0 to 0.031% RBCs were not objectively detectable to the naked eye. Figure 1B and C show a linear correlation between A_414_ measurements and the increase of red/pink colouration in the serial dilutions of RBCs.

### Changes in miRNA levels in artificially haemolysed serum samples

Quantification of sixteen miRNAs was assessed by reverse transcription-quantitative PCR (RT-qPCR) in serum and serum-derived EVs from the nine serial dilution samples (haemolysis series from 0.00% to 0.5% v/v RBCs). We hypothesised that the abundance of specific miRNAs is altered in haemolysed serum samples, while the abundance of others remains stable. This should be similar to reports from several human studies where alterations were the result of contamination with miRNAs enriched in RBCs.

**Fig. 2:**
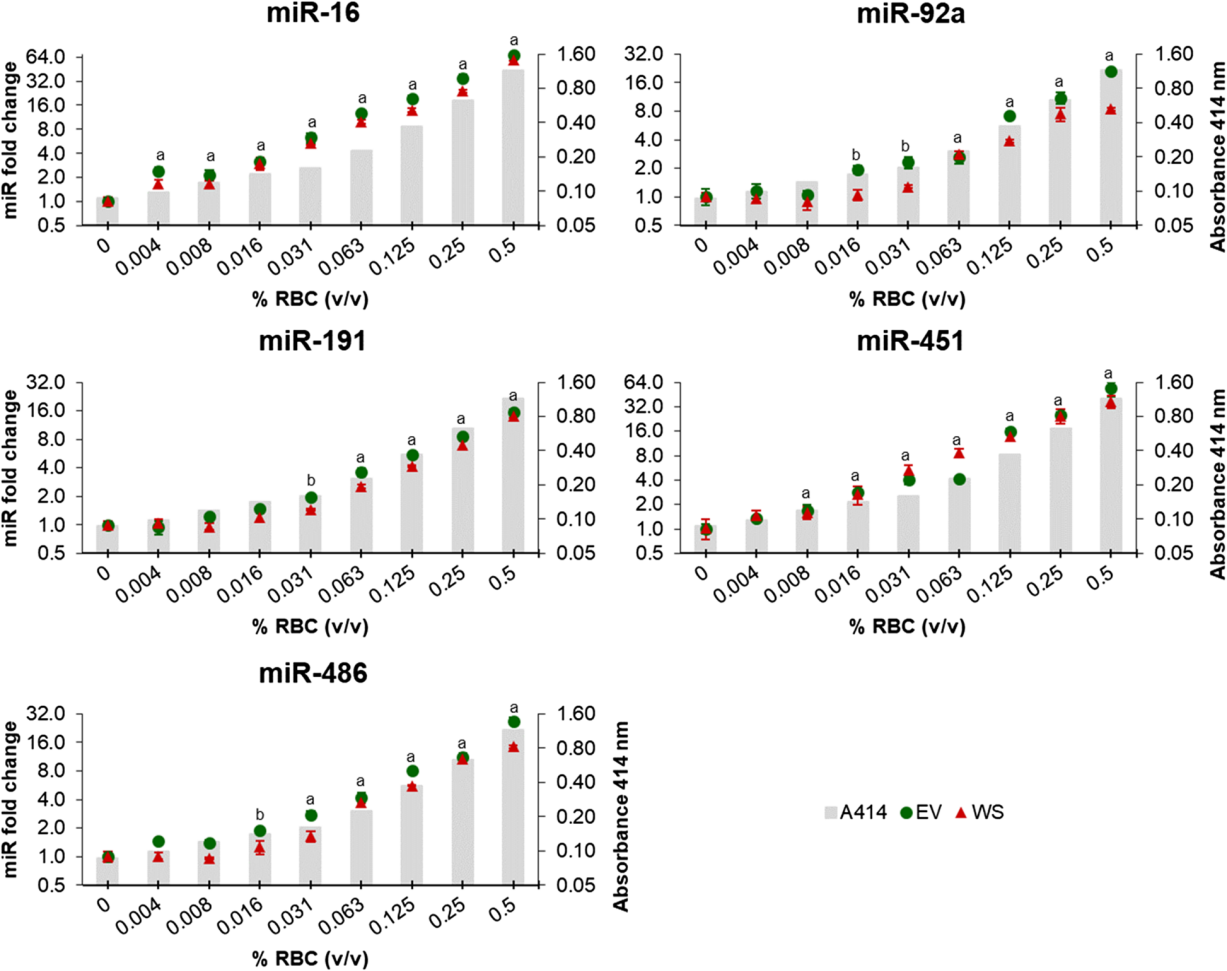
Relative abundance of miRNAs sensitive to haemolysis in whole serum (WS) and serum-derived extracellular vesicles (EV) groups in canine sera from the constructed haemolysis series. Grey columns in the background depict increasing levels of RBCs (% v/v), measured using the haemoglobin absorbance at 414 nm. Fold changes were calculated relative to the non-haemolysed control sample (0.0% RBCs) and normalised using spike-in ssc-miR-292-3p and hsa-miR-934. Experiments were performed in triplicate and markers show the mean ± SD (bars). Statistical significance is represented with an (a) in both groups (WS and EV) and with a (b) in EV group compared to the non-haemolysed control sample (0.0% RBCs). *P* ≤ 0.05, unpaired t-test. RBCs: red blood cells

miR-16, miR-92a, miR-191, miR-451 and miR-486 were all found to be haemolysis-dependent in whole serum (WS group), as well as in serum-derived EVs (EV group), showing similar abundance patterns in both groups (Fig. 2).

A significant increase over 1.5-fold in the abundance of miR-16 was observed in both groups with as little as 0.004% RBCs compared to the non-haemolysed control sample. The same results were found for miR-451, starting from 0.008% RBCs. Increased levels of miR-486 were significant from a concentration of 0.031% RBCs in the WS group and from 0.016% in the EV group compared to the non-haemolysed control sample. Furthermore, significantly higher levels of miR-92a and miR-191 in the WS group were associated with visually detectable haemolysis (≥ 0.063% RBCs), while in the EV group it was also observed in the absence of colour change (Fig. 2).

miR-16 and miR-451 were particularly sensitive to haemolysis in both groups, significantly increasing their abundance despite the lack of visual colour change and to a higher degree in terms of fold changes. As such, at 0.5% RBCs the levels of miR-16 and miR-451 were respectively around 60- and 40-fold higher compared to the non-haemolysed control. In parallel, concentrations of miR-92a, miR-191 and miR-486 were between 9 and 15-fold higher in the WS group or between 15 and 25-fold higher in the EV group compared to the control (Fig. 2).

All five haemolysis-dependent miRNAs (miR-16, miR-92a, miR-191, miR-451 and miR-486) showed a strong positive correlation value (r > 0.95) throughout dilutions of RBC in WS and EV groups, which indicates a linear association between the degree of haemolysis of serum samples and the concentration of these miRNAs (Fig. 2).

**Fig. 3:**
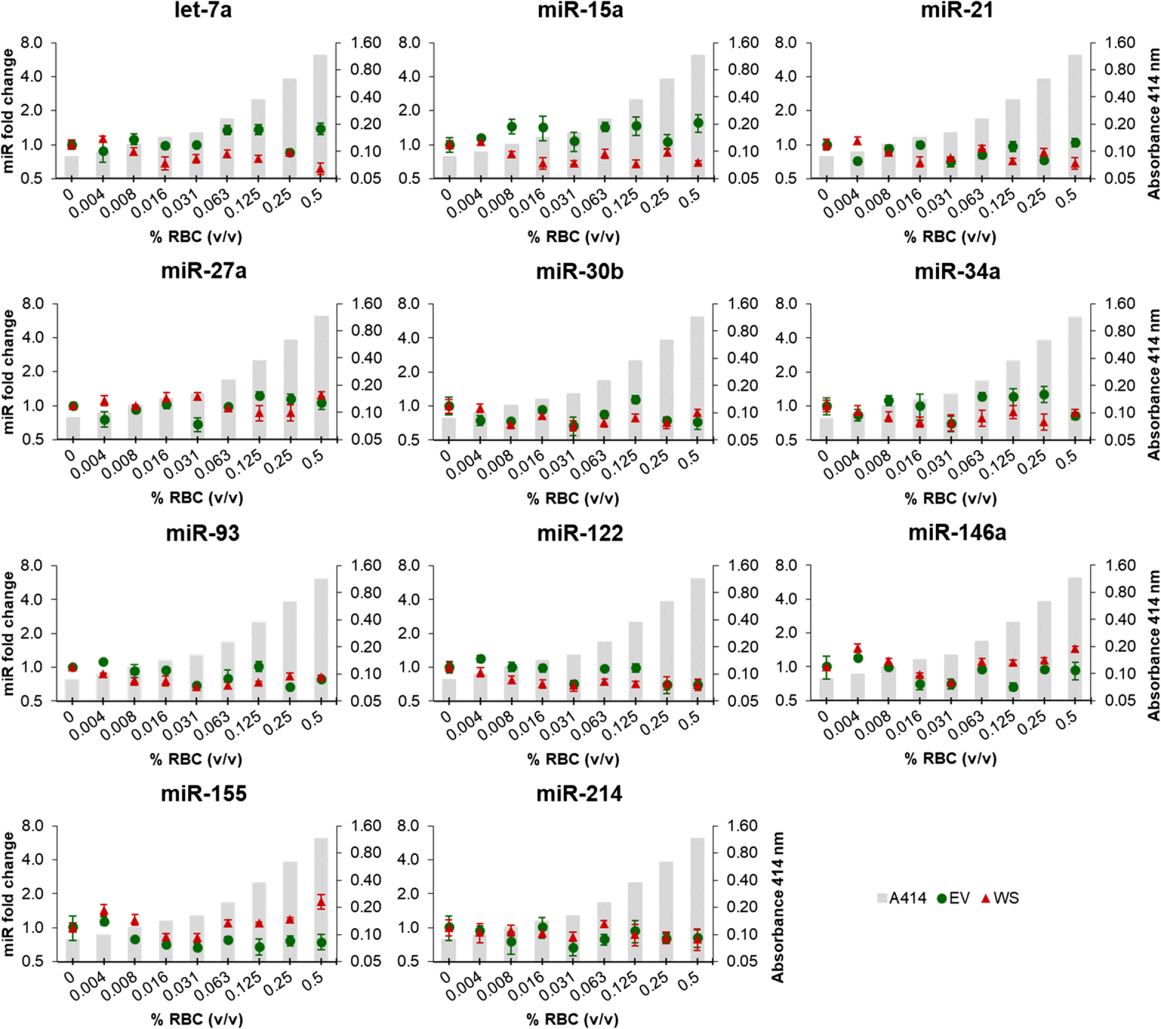
Relative abundance of haemolysis-independent miRNAs in whole serum (WS) and serum-derived extracellular vesicles (EV) groups in canine sera from the constructed haemolysis series. Grey columns in the background depict increasing levels of RBCs (% v/v), measured using the haemoglobin absorbance at 414 nm. Fold changes were calculated relative to the non-haemolysed control sample (0.0% RBCs) and normalised using spike-in ssc-miR-292-3p and hsa-miR-934. Experiments were performed in triplicate and markers show the mean ± SD (bars). No statistical significance compared to the non-haemolysed control sample (0.0% RBCs) was observed in WS and EV groups. *P* ≤ 0.05, unpaired t-test. RBCs: red blood cells

In contrast to all five haemolysis-dependent miRNAs, let-7a, miR-15a, miR-21, miR-27a, miR-30b, miR-34a, miR-93, miR-122, miR-146a, miR-155 and miR-214 showed relatively stable abundance levels in all haemolysis serial dilutions, with no significant variations compared to the non-haemolysed control (Fig. 3).

### Haemolysis and miRNA detection in canine patients´ samples

**Fig. 4:**
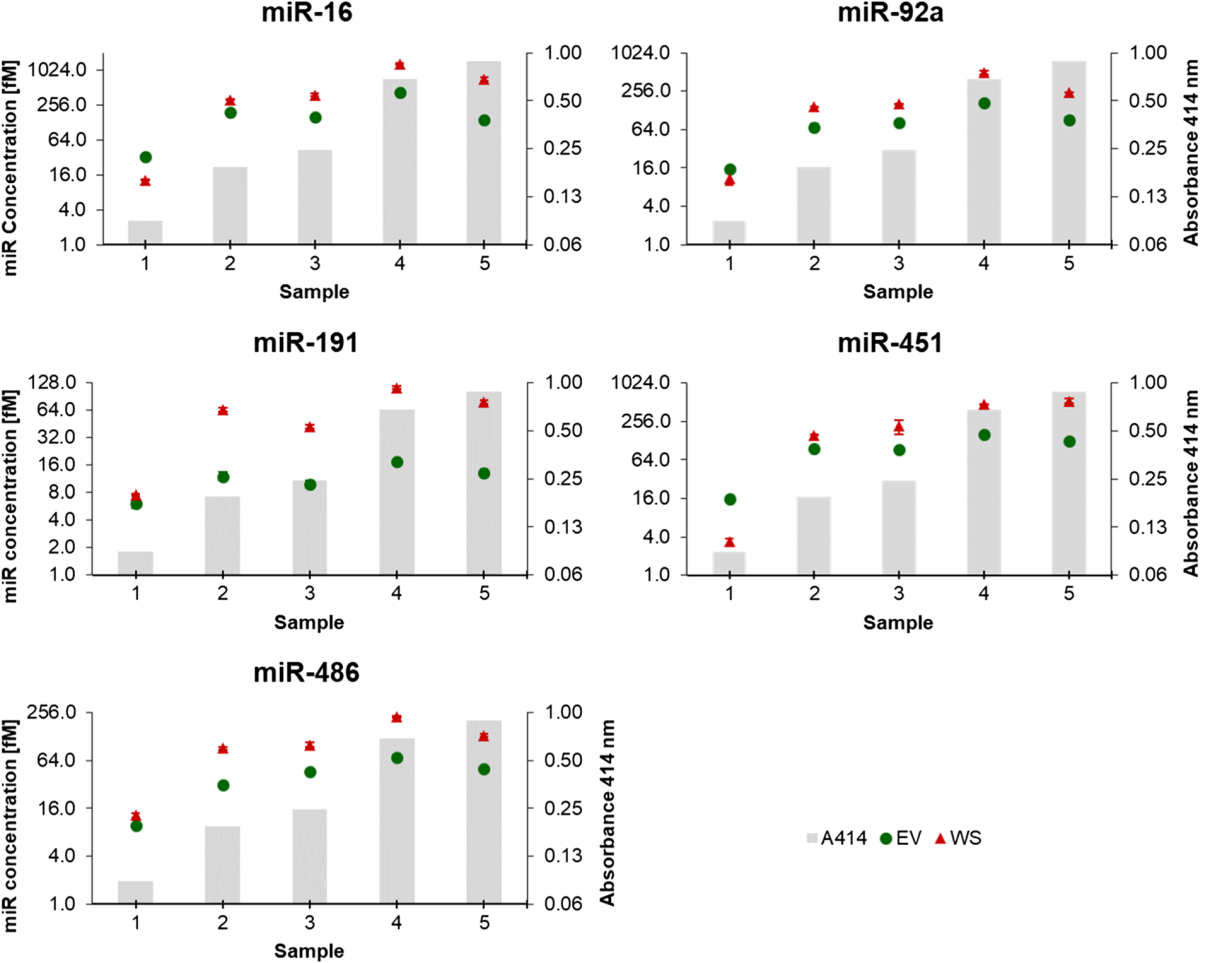
Absolute quantification of miRNAs sensitive to haemolysis in whole serum (WS) and serum-derived extracellular vesicles (EV) groups, in patients’ sera with different degrees of haemolysis (validation experiments). Grey columns in the background depict increasing levels of haemolysis, measured using the haemoglobin absorbance at 414 nm. miRNA concentration was measured by means of absolute quantification, converting Ct values into fM miRNA based on miRNA-specific standard curves. Experiments were performed in triplicate and markers show the mean ± SD (bars)

**Fig. 5:**
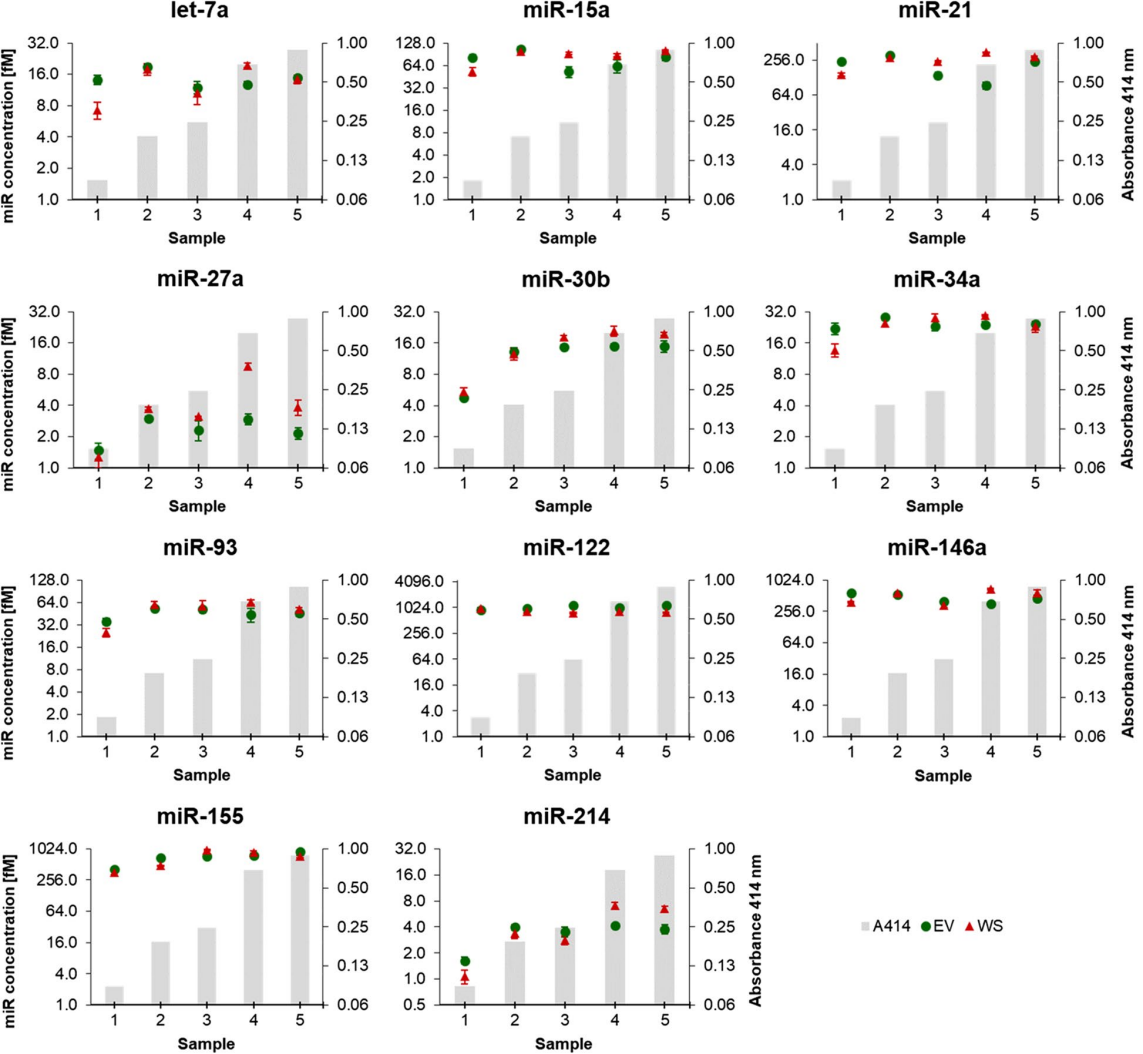
Absolute quantification of haemolysis-independent miRNAs in whole serum (WS) and serum-derived extracellular vesicles (EV) groups, in patients’ sera with different degrees of haemolysis (validation experiments). Grey columns in the background depict increasing levels of haemolysis, measured using the haemoglobin absorbance at 414 nm. miRNA concentration was measured by means of absolute quantification, converting Ct values into fM miRNA based on miRNA-specific standard curves. Experiments were performed in triplicate and markers show the mean ± SD (bars)

For validation of data collected from the artificial haemolysis series, miRNA abundance in samples from non-haemolysed, mildly and strongly haemolysed patients’ sera and serum-derived EVs was investigated (Figs. 4 and 5).

miR-16, miR-92a, miR-191, miR-451 and miR-486, identified as haemolysis-dependent using the artificial haemolysis series, were all found to strongly correlate with the level of haemolysis (individual r values > 0.7) in both WS and EV groups (Fig. 4). The abundance of miR-16 and miR-451 was again found to be particularly dependent on haemolysis, showing increased concentrations of about 100- or 160-fold in strongly haemolysed samples compared to non-haemolysed samples, respectively. The abundance of miR-92, miR-191 and miR-486 was similarly affected by levels of haemolysis, although their quantities in mildly and strongly haemolysed sera was around 10- to 50-fold higher than in non-haemolysed serum (Fig. 4).

The abundance of haemolysis-independent miRNAs let-7a, miR-15a, miR-21, miR-34a, miR-93, miR-122, miR-146a, and miR-155 showed little or no difference in their concentration in all five sera (< 2.5-fold), independent from the levels of haemolysis in WS and EV groups (Fig. 5). However, contrary to the haemolysis series experiments, the abundance of miR-27a, miR-30b, and miR-214 in three single samples showed a different concentration compared to the other samples, but to a lower extent than the haemolysis-dependent miRNAs (Fig. 5). Still, these single variations seem to be unrelated to haemolysis concentration.

## Discussion

During the last decade circulating miRNAs have become one of the most promising, non-invasive, novel biomarkers for many pathologies not only in humans but also in dogs. Several studies have already suggested the use of circulating miRNAs in plasma and serum from dogs as biomarkers for hepatobiliary diseases [13], various canine heart diseases [14–16], lymphoma [17, 18], neoplastic diseases [19], Cushing’s syndrome [20] and liver disease [21, 22], among others. However, many of these studies have not addressed the levels of haemolysis in samples used for their experiments. Haemolysis is caused by the breakdown of RBCs, with the consequent release of haemoglobin and all other intracellular components into the plasma/serum. Specific pathological conditions may induce in vivo haemolysis, yet in vitro haemolysis occurs most commonly and is always a result of suboptimal blood collection or improper sample processing or storage [23]. Thus, release of miRNAs from RBCs upon haemolysis significantly alters the abundance of specific miRNAs in blood, potentially affecting the levels of candidate miRNA biomarkers and the accuracy of serum or plasma-based detection methods [10, 24]. Here we present for the first time in the canine model a systematic analysis of the effects of haemolysis on the abundance of circulating miRNAs. By means of an in vitro controlled haemolysis experiment, we were able to assess changes in miRNA abundance in serum and serum-derived EVs.

Our results showed that concentrations of miR-16, miR-92a, miR-191, miR-451 and miR-486 were significantly influenced by the degree of haemolysis in serum and in serum-derived EVs. Studies performed in human serum and plasma have also described miR-16, mir-451 [9–11, 25], miR-92a [11, 25] and miR-486 [9, 11] as haemolysis-dependent miRNAs, which is not surprising given that these miRNAs were found to be enriched in human RBCs [10, 26]. Likewise, miR-16, miR-92a and miR-451 have been described as one of the most expressed miRNAs in canine erythrocytes [27]. miR-191 in turn has been proposed as a reference miRNA in human non-haemolysed serum [12] and used for normalisation in serum and plasma from dogs [20]. Our data instead showed that the abundance of miR-191 is also dependent on haemolysis levels, although significant increases were associated with visually detectable haemolysis. The abundance of miR-15a and miR-21 has been described as sensitive to haemolysis in human plasma [10], but in contrast, our results indicate a stable concentration in canine serum. We also identified that the abundance of circulating miRNAs previously proposed as biomarkers in dogs are haemolysis-independent: let-7a [28], miR-21, miR-30b [15], miR-34a [18, 28], miR-93 [29], miR-122 [13, 22], miR-155 [28], miR-214 [19] have all been suggested as potential biomarkers in serum, plasma or EVs from dogs. Their relatively stable abundance in our serum samples with varying levels of haemolysis further supports their use as robust miRNAs biomarkers.

We found that the relative abundance of miRNAs in haemolysed and non-haemolysed samples between whole serum (WS) and serum-derived EVs (EV) groups was very similar, which may be explained by two factors. Firstly, mature miRNAs are known to localise in various subcellular compartments, such as vesicles (exosomes, microvesicles) and in association with cytoplasmic proteins [30]. And secondly, although polymer-based precipitation methods are simple, fast, and require no additional equipment for isolation, they enable isolation of mixed EV populations while co-precipitating extracellular proteins, protein complexes, lipoproteins, and nucleoproteins [31, 32]. Therefore, when isolating EVs from haemolysed serum/plasma using polymers, subcellular miRNAs released from RBCs, which are associated with protein complexes or packed in vesicles, may co-precipitate with EVs contained in the sample. A further purification step, such as size-exclusion chromatography (SEC), would allow a reduction of protein contaminants in the final EV isolates [31].

Measuring the absorbance of haemoglobin at 414 nm (A_414_) has been a widely employed method for assessing low to high levels of haemolysis in human plasma and serum samples [9–11, 24, 25]. It is a cost effective and simple method, which requires a minimal sample volume and can be conducted without additional sample processing. Studies investigating human plasma have classified samples as non-haemolysed if the A_414_ value was lower than 0.2 [10, 25]. So far, this has not been assessed in dogs. After measuring A_414_ in visually haemolysed and non-haemolysed canine sera, we observed that A_414_ values below 0.2 were still recognisable as haemolysed under visual inspection. Therefore, we established a lower A_414_ value of 0.1 as cut-off to distinguish non-haemolysed from haemolysed samples in our system. Moreover, as reported in various human studies [10, 12, 24, 25], visual inspection on its own is not sufficient to reliably discriminate haemolysis in serum or plasma, since criteria may vary depending on the expertise of the operator.

The miRNome of human RBCs has been well-studied [33–35], while investigations in dogs at this level have not yet been reported. One study partially analysed the canine miRNome [27]; however, an entire miRNA-sequencing analysis in RBCs is still needed. This may predict changes in haemolysed samples and thus avoid misinterpretation of miRNA abundance in serum or plasma.

## Conclusion

Our results represent the first methodological approach to assess the effects of haemolysis on circulating miRNAs not only in whole canine serum samples but also in canine serum-derived EVs. This provides new data for further circulating biomarker discovery. Although this study was limited to a relatively small sample size, our targeted approach identified that haemolysis dramatically influences the abundance of certain circulating miRNAs, while others remain relatively independent of the levels of haemolysis. Moreover, we found differences from studies in human samples, which suggests a species-specific variation. Since the entire canine RBCs miRNome is still unknown, our data suggest that future research on canine circulating miRNA should assess haemolysis before introducing candidate biomarkers and therefore assure accuracy of serum or plasma-based assays.

## Methods

### Sample collection

Serum samples were all collected from residual specimens following routine diagnostic laboratory tests (post treatment controls) in dogs presented at the Small Animal Clinic, Department of Veterinary Medicine at the Freie Universität Berlin. Blood samples were collected and allowed to clot at room temperature. After coagulation, serum was separated from the blood cells by centrifugation at 2000 × *g* for 10 min at 20 °C and within 2 h frozen at -20 °C. In order to obtain a RBC concentrate for induction of artificial haemolysis, whole blood collected in K_2_EDTA from one patient was centrifuged at 2000 × *g* for 10 min at 20 °C and plasma and buffy coat were discarded. RBCs were stored at -20 °C until use.

### Haemolysis series and haemolysis assessment

A pool of non-haemolysed serum samples (*n* = 12) was used for the construction of a haemolysis series and a control sample. In addition, five randomly selected patients’ sera with different degrees of haemolysis were employed to validate the results obtained from haemolysis-induced experiments. The degree of haemolysis was assessed in all samples by measuring the absorbance of haemoglobin at 414 nm (A_414_), using water as blank [10, 36], with the NanoDrop™ 1000 spectrophotometer (Thermo Fisher Scientific, Inc., Waltham, MA, USA). Absorbance measurements were performed in triplicate.

Sera intended for preparation of the non-haemolysed pool sample were pre-selected via visual inspection. Pooled serum samples were ultimately selected based on an A_414_ < 0.1, which represents a *bona fide* value for non-haemolysed serum or plasma samples, lower than previously reported in the human system [11, 12, 25, 36]. Haemolysis was induced in vitro by adding a known volume of lysed RBCs into the pool of non-haemolysed sera and mixing vigorously using a vortex. From this initial dilution, seven 1:2 serial dilutions were prepared, starting from 0.5% to 0.00% v/v, which originated a haemolysis series of nine samples, including the non-haemolysed sample control (Fig. 1A).

Serum samples from five randomly selected patients were categorized according to the haemolysis degree in non-haemolysed (A_414_ = 0.088), mildly haemolysed (A_414_ = 0.192 and 0.245), and strongly haemolysed (A_414_ = 0.682 and 0.885).

### Isolation of extracellular vesicles

EVs were isolated from 300 µl serum using a polymer-based precipitation reagent kit (Total Exosome Isolation Reagent - from serum, Cat#4478360, Invitrogen, Vilnius, Lithuania) following the manufacturer’s protocol. EV pellets were resuspended in 300 µl PBS, which represented the original volume of serum. In our previous published study [37], using this commercial kit and same isolation protocol, we assessed size distribution, concentration, morphology and CD63 expression in EVs (e.g. exosomes), which validated this isolation procedure from small volumes of canine serum samples.

### RNA isolation and RT-qPCR

RNA isolation from serum was carried out from two types of samples: whole serum (WS) and serum-derived EVs (EV). RNA was extracted from 300 µl serum and EVs-suspension using 3 volumes of TRIzol™ Reagent (Invitrogen, Carlsbad, CA, USA) supplemented with acetic acid (125 mM), according to the manufacturer’s instructions. 250 fmol of two synthetic miRNAs, ssc-miR-292-3p [38] and hsa-miR-934 [39], which are not expressed in dogs, were spiked-in after 5 min of incubation in TRIzol™ Reagent. Following separation by chloroform, 6 µg glycogen (R0551, Thermo Fisher Scientific) was added as RNA co-precipitant to the aqueous phase. RNA was eluted in 50 µl nuclease-free water.

Sixteen known *Canis familiaris* miRNAs were selected for evaluation, on the basis that they had already been reported as haemolysis-dependent or -independent miRNAs in the human system, or have been previously proposed as diagnostic/prognostic biomarkers in dogs (Additional file 1).

The quantification of miRNAs through RT‐qPCR was performed using the highly sensitive and specific miR-Q assay, as previously described [40, 41], with some modifications in the RT protocol: a fixed amount of 3.6 µl RNA template was reverse transcribed into cDNA and 3 specific RT-miRNA primers were multiplexed per RT reaction in a final volume mix of 12 µl. The abundance of miRNAs in samples from the haemolysis series was normalised using spike-in ssc-miR-292-3p and hsa-miR-934. Measurements were performed in triplicate, based on the 2‐ΔΔCT method [42] and following protocols detailed before [41]. The concentration of miRNAs in patients’ samples used for validation was measured in triplicate by means of absolute quantification, converting Ct values into fM miRNA based on miRNA-specific standard curves [40]. The entire set of oligonucleotides used in this study is provided in Tables 1–3 (Additional file 2). miRNA primers were designed as reported before [40]. All oligonucleotides were synthesised by Sigma‐Aldrich (Darmstadt, Germany).

### Statistical analysis

A two-tailed Student’s t-test was used for comparison between the non-haemolysed control group and the samples containing the serial dilutions of RBCs. Results are expressed as means of triplicate measurements (technical replicates) ± standard deviation (SD). A *P*-value < 0.05 was considered statistically significant. Pearson correlation coefficient (r) was calculated to determine the linear association between % RBC and increase in the miRNA abundance.

## Supplementary Information

**Additional file 1. **Literature review of targeted miRNAs of this study. Human and dog-based studies indicating an association of miRNAs with haemolysis or suggesting their use as candidate biomarkers. 

**Additional file 2: Table 1.** lists all primer sequences of selected miRNAs. **Table 2.** lists the synthetic RNA sequences of spiked miRNAs. **Table 3.** lists the synthetic cDNA sequences used for constructing miRNA-specific qPCR standard curves. 

## Data Availability

All data generated or analysed during this study are included in this published article.

## Abbreviations

A_414_: Absorbance of haemoglobin at 414 nm
EVs: Extracellular vesicles
fM: Femtomolar
miRNAs: MicroRNAs
RBCs: Red blood cells
RT‐qPCR: Reverse transcription-quantitative PCR
SD: Standard deviation
SEC: Size-exclusion chromatography
