# Supplementary material for: Systematic analysis of different degrees of haemolysis on miRNA levels in serum and serum-derived extracellular vesicles from dogs
Source: BMC Vet Res. 2022 Sep 22;18:355. doi: 10.1186/s12917-022-03445-8 (PMC9494854; doi:10.1186/s12917-022-03445-8)
Supplement: Supplementary file 1 — Additional file 1. Literature review of targeted miRNAs of this study. Human and dog-based studies indicating an association of miRNAs with haemolysis or suggesting their use as candidate biomarkers. [file 12917_2022_3445_MOESM1_ESM.docx]

**Additional file 1. Literature review of targeted miRNAs of this study.** Human and dog-based studies indicating an association of miRNAs with haemolysis or suggesting their use as candidate biomarkers.

| Nr | miRNA | Reference |
| --- | --- | --- |
| 1 | let-7a | Blondal et al. (2013); Kirschner et al. (2013); Elshafie et al. (2021) |
| 2 | miR-15a | Kirschner et al. (2011); Blondal et al. (2013); Kirschner et al. (2013) |
| 3 | miR-16 | Kirschner et al. (2011); Blondal et al. (2013); Kirschner et al. (2013); Pizzamiglio et al. (2017) |
| 4 | miR-21 | Kirschner et al. (2013); Hulanicka et al. (2014) |
| 5 | miR-27a | Blondal et al. (2013); MacLellan et al. (2014); Craig et al. (2019) |
| 6 | miR-30b | Blondal et al. (2013); Hulanicka et al. (2014) |
| 7 | miR-34a | Craig et al. (2019); Elshafie et al. (2021) |
| 8 | miR-92a | Kirschner et al. (2011); Fujiwara-Igarashi et al. (2015); Pizzamiglio et al. (2017) |
| 9 | miR-93 | Myklebust et al. (2019); Garnica et al. (2020) |
| 10 | miR-122 | Koenig et al. (2016); Oosthuyzen et al. (2018); Sanders et al. (2021) |
| 11 | miR-146a | Blondal et al. (2013); Kirschner et al. (2013) |
| 12 | miR-155 | Kirschner et al. (2011); Elshafie et al. (2021) |
| 13 | miR-191 | Blondal et al. (2013); Myklebust et al. (2019); Sanders et al. (2021) |
| 14 | miR-214 | Heishima et al. (2017) |
| 15 | miR-451 | Kirschner et al. (2011); Blondal et al. (2013); Kirschner et al. (2013); Pizzamiglio et al. (2017) |
| 16 | miR-486 | Blondal et al. (2013); Kirschner et al. (2013); Pizzamiglio et al. (2017) |

**References**

Blondal, T., Nielsen, S.J., Baker, A., Andreasen, D., Mouritzen, P., Teilum, M.W., and Dahlsveen, I.K. (2013). Assessing sample and miRNA profile quality in serum and plasma or other biofluids. *Methods* **59**: S1-S6.

Craig, K.K., Wood, G.A., Keller, S.M., Mutsaers, A.J., and Wood, R.D. (2019). MicroRNA profiling in canine multicentric lymphoma. *PloS one* **14**: e0226357.

Elshafie, N.O., Nascimento, N.C.D., Lichti, N.I., Kasinski, A.L., Childress, M.O., and Santos, A.P.D. (2021). MicroRNA Biomarkers in Canine Diffuse Large B-Cell Lymphoma. *Veterinary Pathology* **58**: 34-41.

Fujiwara-Igarashi, A., Igarashi, H., Mizutani, N., Goto-Koshino, Y., Takahashi, M., Ohno, K., and Tsujimoto, H. (2015). Expression profile of circulating serum microRNAs in dogs with lymphoma. *The Veterinary Journal* **205**: 317-321.

Garnica, T.K., Lesbon, J.C., Ávila, A.C., Rochetti, A.L., Matiz, O.R., Ribeiro, R., Zoppa, A., Nishiya, A.T., Costa, M.T., and De Nardi, A.B. (2020). Liquid biopsy based on small extracellular vesicles predicts chemotherapy response of canine multicentric lymphomas. *Scientific reports* **10**: 1-11.

Heishima, K., Ichikawa, Y., Yoshida, K., Iwasaki, R., Sakai, H., Nakagawa, T., Tanaka, Y., Hoshino, Y., Okamura, Y., and Murakami, M. (2017). Circulating microRNA-214 and-126 as potential biomarkers for canine neoplastic disease. *Scientific reports* **7**: 1-14.

Hulanicka, M., Garncarz, M., Parzeniecka-Jaworska, M., and Jank, M. (2014). Plasma miRNAs as potential biomarkers of chronic degenerative valvular disease in Dachshunds. *BMC veterinary research* **10**: 1-8.

Kirschner, M.B., Edelman, J.J.B., Kao, S.C.-H., Vallely, M.P., Van Zandwijk, N., and Reid, G. (2013). The impact of hemolysis on cell-free microRNA biomarkers. *Frontiers in genetics* **4**: 94.

Kirschner, M.B., Kao, S.C., Edelman, J.J., Armstrong, N.J., Vallely, M.P., Van Zandwijk, N., and Reid, G. (2011). Haemolysis during sample preparation alters microRNA content of plasma. *PloS one* **6**: e24145.

Koenig, E.M., Fisher, C., Bernard, H., Wolenski, F.S., Gerrein, J., Carsillo, M., Gallacher, M., Tse, A., Peters, R., and Smith, A. (2016). The beagle dog MicroRNA tissue atlas: identifying translatable biomarkers of organ toxicity. *BMC genomics* **17**: 1-13.

Maclellan, S.A., Macaulay, C., Lam, S., and Garnis, C. (2014). Pre-profiling factors influencing serum microRNA levels. *BMC clinical pathology* **14**: 1-11.

Myklebust, M.P., Rosenlund, B., Gjengstø, P., Bercea, B.S., Karlsdottir, Á., Brydøy, M., and Dahl, O. (2019). Quantitative PCR measurement of miR-371a-3p and miR-372-p is influenced by hemolysis. *Frontiers in genetics* **10**: 463.

Oosthuyzen, W., Ten Berg, P., Francis, B., Campbell, S., Macklin, V., Milne, E., Gow, A., Fisher, C., Mellanby, R., and Dear, J. (2018). Sensitivity and specificity of microRNA‐122 for liver disease in dogs. *Journal of veterinary internal medicine* **32**: 1637-1644.

Pizzamiglio, S., Zanutto, S., Ciniselli, C.M., Belfiore, A., Bottelli, S., Gariboldi, M., and Verderio, P. (2017). A methodological procedure for evaluating the impact of hemolysis on circulating microRNAs. *Oncology letters* **13**: 315-320.

Sanders, K., Veldhuizen, A., Kooistra, H.S., Slob, A., Timmermans-Sprang, E.P., Riemers, F.M., Daminet, S., Fracassi, F., Van Nimwegen, S.A., and Meij, B.P. (2021). Circulating MicroRNAs as Non-invasive Biomarkers for Canine Cushing's Syndrome. *Frontiers in Veterinary Science* **8**.
