# Supplementary material for: Systematic analysis of different degrees of haemolysis on miRNA levels in serum and serum-derived extracellular vesicles from dogs
Source: BMC Vet Res. 2022 Sep 22;18:355. doi: 10.1186/s12917-022-03445-8 (PMC9494854; doi:10.1186/s12917-022-03445-8)
Supplement: Supplementary file 2 — Additional file 2: Table 1. lists all primer sequences of selected miRNAs. Table 2. lists the synthetic RNA sequences of spiked miRNAs. Table 3. lists the synthetic cDNA sequences used for constructing miRNA-specific qPCR standard curves. [file 12917_2022_3445_MOESM2_ESM.docx]

**Table 1. Primer sequences of selected miRNAs.** Genes for normalisation are presented in Italic.

| Nr | miRNA | RT6-miRNA (5'->3') | miRNA-rev (5'->3') |
| --- | --- | --- | --- |
| 1 | let-7a | TGTCAGGCAACCGTATTCACCGTGAGTGGTAACTAT | CGTCAGATGTCCGAGTAGAGGGGGAACGGCGTGAGGTAGTAGGTTGTATA |
| 2 | miR-15a | TGTCAGGCAACCGTATTCACCGTGAGTGGTACAAAC | CGTCAGATGTCCGAGTAGAGGGGGAACGGCGTAGCAGCACATAATGGTT |
| 3 | miR-16 | TGTCAGGCAACCGTATTCACCGTGAGTGGTCGCCAA | CGTCAGATGTCCGAGTAGAGGGGGAACGGCGTAGCAGCACGTAAATA |
| 4 | miR-21 | TGTCAGGCAACCGTATTCACCGTGAGTGGTTCAACA | CGTCAGATGTCCGAGTAGAGGGGGAACGGCGTAGCTTATCAGACTGA |
| 5 | miR-27a | TGTCAGGCAACCGTATTCACCGTGAGTGGTGCGGAAC | CGTCAGATGTCCGAGTAGAGGGGGAACGGCGTTCACAGTGGCTAAG |
| 6 | miR-30b | TGTCAGGCAACCGTATTCACCGTGAGTGGTAGCTGA | CGTCAGATGTCCGAGTAGAGGGGGAACGGCGTGTAAACATCCTACACTCA |
| 7 | miR-34a | TGTCAGGCAACCGTATTCACCGTGAGTGGTACAACC | CGTCAGATGTCCGAGTAGAGGGGGAACGGCGTGGCAGTGTCTTAGCTGGT |
| 8 | miR-92a | TGTCAGGCAACCGTATTCACCGTGAGTGGTACAGGC | CGTCAGATGTCCGAGTAGAGGGGGAACGGCGTATTGCACTTGTCCCGGCC |
| 9 | miR-93 | TGTCAGGCAACCGTATTCACCGTGAGTGGTCTACCT | CGTCAGATGTCCGAGTAGAGGGGGAACGGCGTCAAAGTGCTGTTCGTG |
| 10 | miR-122 | TGTCAGGCAACCGTATTCACCGTGAGTGGTCAAACA | CGTCAGATGTCCGAGTAGAGGGGGAACGGCGTTGGAGTGTGACAATGGTGT |
| 11 | miR-146a | TGTCAGGCAACCGTATTCACCGTGAGTGGTAACCCA | CGTCAGATGTCCGAGTAGAGGGGGAACGGCGTGAGAACTGAATTCCATGG |
| 12 | miR-155 | TGTCAGGCAACCGTATTCACCGTGAGTGGTACCCCT | CGTCAGATGTCCGAGTAGAGGGGGAACGGCGTTAATGCTAATCGTGATAGG |
| 13 | miR-191 | TGTCAGGCAACCGTATTCACCGTGAGTGGTAGCTGC | CGTCAGATGTCCGAGTAGAGGGGGAACGGCGCAACGGAATCCCAAAA |
| 14 | miR-214 | TGTCAGGCAACCGTATTCACCGTGAGTGGTACTGCC | CGTCAGATGTCCGAGTAGAGGGGGAACGGCGACAGCAGGCACAGACA |
| 15 | miR-451 | TGTCAGGCAACCGTATTCACCGTGAGTGGTAACTCA | CGTCAGATGTCCGAGTAGAGGGGGAACGGCGAAACCGTTACCATTACTGA |
| 16 | miR-486 | TGTCAGGCAACCGTATTCACCGTGAGTGGTTCGGGG | CGTCAGATGTCCGAGTAGAGGGGGAACGGCGTCCTGTACTGAGCTGC |
| 17 | *ssc-miR-292-3p* | *GTCAGGCAACCGTATTCACCGTGAGTGGTTCG AAA* | *CGTCAGATGTCCGAGTAGAGGGGGAACGGCGGGTGTTTCCGCCCGGTTT* |
| 18 | *hsa-miR-934* | *GTCAGGCAACCGTATTCACCGTGAGTGGTCCAGTG* | *CGTCAGATGTCCGAGTAGAGGGGGAACGGCGTTGTCTACTACTGGAGA* |

**Table 2. Synthetic RNA sequences of spiked miRNAs.**

| Nr | miRNA | Sequence (5'->3') |
| --- | --- | --- |
| 1 | ssc-miR-292-3p | GGUGUUUCCGCCCGGUUUCGA |
| 2 | hsa-miR-934 | UGUCUACUACUGGAGACACUGG |

**Table 3. Synthetic cDNA sequences employed for constructing miRNA-specific qPCR standard curves.**

| Nr | miRNA | cDNA-sequence (5'->3') |
| --- | --- | --- |
| 1 | let-7a | TGTCAGGCAACCGTATTCACCGTGAGTGGTAACTATACAACCTACTACCTCA |
| 2 | miR-15a | TGTCAGGCAACCGTATTCACCGTGAGTGGTACAAACCATTATGTGCTGCTA |
| 3 | miR-16 | TGTCAGGCAACCGTATTCACCGTGAGTGGTCGCCAATATTTACGTGCTGCTA |
| 4 | miR-21 | TGTCAGGCAACCGTATTCACCGTGAGTGGTAGTCAACATCAGTCTGATAAGCTA |
| 5 | miR-27a | TGTCAGGCAACCGTATTCACCGTGAGTGGTCGGAACTTAGCCACTGTGAA |
| 6 | miR-30b | TGTCAGGCAACCGTATTCACCGTGAGTGGTAGCTGAGTGTAGGATGTTTACA |
| 7 | miR-34a | TGTCAGGCAACCGTATTCACCGTGAGTGGTACAACCAGCTAAGACACTGCCA |
| 8 | miR-92a | TGTCAGGCAACCGTATTCACCGTGAGTGGTACAGGCCGGGACAAGTGCAATA |
| 9 | miR-93 | TGTCAGGCAACCGTATTCACCGTGAGTGGTCTACCTGCACGAACAGCACTTTG |
| 10 | miR-122 | TGTCAGGCAACCGTATTCACCGTGAGTGGTCAAACACCATTGTCACACTCCA |
| 11 | miR-146a | TGTCAGGCAACCGTATTCACCGTGAGTGGTAACCCATGGAATTCAGTTCTCA |
| 12 | miR-155 | TGTCAGGCAACCGTATTCACCGTGAGTGGTACCCCTATCACGATTAGCATTAA |
| 13 | miR-191 | TGTCAGGCAACCGTATTCACCGTGAGTGGTAGCTGCTTTTGGGATTCCGTTG |
| 14 | miR-214 | TGTCAGGCAACCGTATTCACCGTGAGTGGTACTGCCTGTCTGTGCCTGCTGT |
| 15 | miR-451 | TGTCAGGCAACCGTATTCACCGTGAGTGGTAACTCAGTAATGGTAACGGTTT |
| 16 | miR-486 | TGTCAGGCAACCGTATTCACCGTGAGTGGTTCGGGGCAGCTCAGTACAGGA |
